# Prospective for urban informatics

**DOI:** 10.1007/s44212-022-00006-0

**Published:** 2022-09-09

**Authors:** Wenzhong Shi, Michael Goodchild, Michael Batty, Qingquan Li, Xintao Liu, Anshu Zhang

**Affiliations:** 1 Otto Poon Charitable Foundation Smart Cities Research Institute and Department of Land Surveying and Geo-Informatics, The Hong Kong Polytechnic University, Hong Kong, China; 2 University of California, Santa Barbara, Santa Barbara, USA; 3 Centre for Advanced Spatial Analysis, University College London, London, UK; 4 Guangdong Laboratory of Artificial Intelligence and Digital Economy (Shenzhen), Shenzhen University, Shenzhen, China

**Keywords:** Urban informatics, Urban science, Urban computing, Urban sensing, Smart cities

## Abstract

The specialization of different urban sectors, theories, and technologies and their confluence in city development have led to a greatly accelerated growth in urban informatics, the transdisciplinary field for understanding and developing the city through new information technologies. While this young and highly promising field has attracted multiple reviews of its advances and outlook for its future, it would be instructive to probe further into the research initiatives of this rapidly evolving field, to provide reference to the development of not only urban informatics, but moreover the future of cities as a whole. This article thus presents a collection of research initiatives for urban informatics, based on the reviews of the state of the art in this field. The initiatives cover three levels, namely the future of urban science; core enabling technologies including geospatial artificial intelligence, high-definition mapping, quantum computing, artificial intelligence and the internet of things (AIoT), digital twins, explainable artificial intelligence, distributed machine learning, privacy-preserving deep learning, and applications in urban design and planning, transport, location-based services, and the metaverse, together with a discussion of algorithmic and data-driven approaches. The article concludes with hopes for the future development of urban informatics and focusses on the balance between our ever-increasing reliance on technology and important societal concerns.

## Introduction

Facing the increasing specialization of contemporary urban sectors, theories, and technologies, transdisciplinary perspectives are clearly necessary to synergize these numerous fields into the wider mission of developing more efficient and livable cities. Such a transdisciplinary approach for understanding and developing the city through new information technologies, urban informatics becomes “particularly timely” (Shi et al. 2021, p. 1) and has experienced a dramatic growth in the second decade of the twenty-first century.

As a young field of immense potential, urban informatics has attracted considerable efforts on summarizing its advances, as well as anticipating its future developments and accompanying societal concerns. *The Handbook of Research on Urban *Informatics (Foth 2008) emphasized public participation and engagement of urban communities, as well as selected technologies including navigation, virtual cities, wireless infrastructures, and mobile applications. The last chapters of the book elaborated an outlook for urban informatics based on a limited number of forward-looking technologies at that time as well as social and cultural concerns. The technologies and applications in more fields pertaining to urban informatics have continually been updated in various proceedings such as that of the 2014 Workshop on Big Data and Urban Informatics (Thakuriah et al. 2017a). The innovations and challenges from technical and political-economic perspectives in the use of big data for urban informatics were discussed in that book (Thakuriah et al. 2017b) serving to widen the field. The role of urban informatics in supporting technologies of planning was further elaborated by journal special issues, such as *Planning Support Science with Urban Informatics* in *Environment and Planning B: Urban Analytics and City Science* (Pan et al. 2020). The theories, technologies, and applications in this field have been systematically expounded in the recent book *Urban Informatics* (Shi et al. 2021) where five dimensions were defined: urban science, systems and applications, sensing, big data infrastructure, and computing. The epilogue of the book probed into multiple alternative visions for urban informatics, cautioning for possible unintended consequences and calling for a continued exploration of the broader impacts of urban informatics (Goodchild 2021a).

In this first issue of the international journal *Urban Informatics* which is dedicated to sketching the outline of the field, it would be meaningful to again catch up on the accelerated evolution of this field, to probe into its research initiatives which could serve as reference to the development of not only this field, but the future cities as a whole. The research initiatives concern three closely intercorrelated aspects, namely future urban science, backbone technologies, and urban applications. Deeper understanding of cities and more powerful applications of urban design, management, and services are enabled by cutting-edge technologies in urban sensing, computing, and big data infrastructure. These technologies include, for example, geospatial artificial intelligence (GeoAI), high-definition (HD) map, quantum computing, AI and the IoT (artificial intelligence and the internet of things, abbreviated to AIoT), digital twins, and approaches involving deep learning and machine learning in distributed, explicable, and privacy-preserving approaches to the acquisition and analysis of big data. In turn, urban science and applications not only motivate technological progress, but also reveal the wider impacts of these technologies and their subsequent applications, resulting in thinking about the most appropriate guidance in technological developments that hopefully will realize the greater good.

In the rest of this article, Sect. 2 will briefly review the evolving definitions of urban informatics, how it has emerged as a disciplinary focus, as well as the state of the art of this field. Sect. 3 will discuss the research initiatives of urban informatics at three levels, from new advances in urban science and a selection of forward-looking core technologies, to core application fields involving future cities and citizen lives and precautions facing the inevitable advent of an algorithmic and data-driven era. Sect. 4 will present our concluding remarks, pointing out what is not covered by the journal at least so far and our hopes for the future development of the field of urban informatics.

## The state of the art of urban informatics

### The rise of urban informatics

As early as 1987, under the term “urban informatics planning”, Hepworth (1987) discussed the research and policies for promoting and managing the development of metropolitan area which were undergoing the transformations posed by the “information revolution”. This envisioning discussion may be also seen as an outlook on the comprehensive integration of informatics with urban planning and development. Townsend (2008) offered two definitions of urban informatics in the book devoted to this topic (Foth 2008). He defined it as: “the collection, classification, storage, retrieval, and dissemination of recorded knowledge of, relating to, characteristic of, or constituting a city” and “the collection, classification, storage, retrieval, and dissemination of recorded knowledge in a city” (p. xxiii). The book emphasizes the role of urban informatics in the real-time examination of the real-time urban systems, and substantially involved related location-sensitive technologies, such as navigation, wireless communications, and mobile applications. At that stage, geomatics seemed mainly utilized as the enabling technologies under the “informatics” aspect of urban informatics. Batty (2013) stressed the importance of “big data” obtained by densely embedded sensors in cities and geospatial modelling for urban informatics. This drew more attention to the spatial aspects and geomatics in urban informatics, the latter concerning the measurement of urban objects and management of the resultant data.

Different urban sectors have been more specialized together with their underlying scientific disciplines, making it more frequently necessary to solve ever more complex urban issues by synergizing the possibly contradictory perspectives from multiple sectors. Shi et al. (2021) thus gave more attention to the interdisciplinary nature of urban informatics and defined this field as “an interdisciplinary approach to understanding, managing, and designing the city using systematic theories and methods based on new information technologies” (p. 2). They proposed urban science, informatics, and geomatics as three pillars of urban informatics, among which urban science studies urban activities, places, and flows; geomatics focusses on the sensing of urban objects and management of the massive and complex urban-sensing data; and informatics enables information handling and computation to develop more capable and intelligent urban applications.

Compared with the fields associated with individual urban sectors, such as demography, economy, transportation, land use, retail, and energy sources, urban informatics is distinctive in that it takes computational methods and models as a core to understand urban phenomena and subsequently to work out solutions to corresponding urban issues (Shi et al. 2021). By capturing and learning from multisource and heterogeneous urban-sensing data, sophisticated models and powerful computation tools may decompose complex urban phenomena to the ones that are easier to be understood and reasoned by domain human experts. On the other hand, dynamics and interests of multiple sectors may be fused in high-dimensional models and multi-objective optimizations working on the urban-sensing data to reach better compromised solutions between different urban sectors and stakeholders. In this way, urban informatics can both advance the theoretical development of other urban-related disciplines and generate better solutions to many urban issues than the disciplines focusing on individual urban sectors. Cutting-edge science and technologies for capturing urban-sensing data are the bases to get the materials for computation in urban informatics. Such importance of geomatics for urban informatics may explain the difference between urban informatics and urban science or its integration with computer science. Meanwhile, geomatics or geoinformatics in the urban context, which may be referred to as urban geomatics or geoinformatics, do not necessarily rely on new information and computing technologies.

### Urban science

Urban science first emerged hard on the heels of the Newtonian revolution in mechanics at the beginning of the industrial revolution in Europe. Several commentators suggests that classic physical concepts such as gravitational force and potential might be used to understand social forces particularly those associated with distance whose effects were radically changing through the developments of new modes of rapid travel such as the railways. But it was not until the middle of the lasmeizhout century that a sustained development of formal models of cities emerged, building on developments in urban economics and location theory (Isard 1956). At the same time, land use and transportation modelling provided the rapid momentum for applications in practice which were the earliest examples of computer models of cities. After this first wave of what we now loosely call ‘urban science’, another began focusing more theoretically on dynamics (Wilson 1981) and in the last 20 years this has evolved an economic basis for urban size and shape, particularly with respect to fractal morphologies, urban agglomeration and allometry (Bettencourt 2021).

We need to note however that urban science and its analog in the form of a science of cities represent a recent shorthand for many movements developing systematic and computable urban theories of how cities form and evolve (Batty 2023). An important qualification which is important to our arguments here is that urban science is a science of complexity revolting around geometry and relationships between populations and the built environment; we do not use the term to cover the science of urban ecology, building physics, pollution and so on which lie on the edge of the science that urban informatics relates to.

In recent years, urban science has progressed from looking into questions where place, space, and location define the structure of the city to various networks that link locations together through the critical flows that determine how the city glues itself together as various kinds of energy (Batty 2021). Under the perennial core topics in urban science such as the urban morphology and scaling, theoretical developments have evolved along trends in the development of cities and human society. For example, new ideas extending urban scaling laws to the third dimension, and analysis of critical densities above which cities shift to vertical growth have been identified (Molinero & Thurner 2021), while human-centered perspectives incorporating people’s subjective perceptions in environmental and socioeconomic contexts reflected in human dynamics (Shaw & Sui 2020) have been stressed.

Models which build on ideas about the key signatures defining the structure of cities in terms of scale, size and density (Batty 2008) link more traditional approaches in urban economics to models of land use and transportation behavior. This new urban science attempts a more synoptic view of how urban phenomena can be treated in aggregate and disaggregate terms as well as in static and dynamic equilibria. These form key elements in defining an urban science, a science of cities that is focused on integrating many of the physical, economic, and social foci that define a much wider range of approaches in urban studies.

Massive high-frequency data streams, captured through lightweight sensors embedded in every corner of the city, are enabling us to capture more and more features of the city which are leading to many new urban theories and the development of many new tools and models. The smart city is at the forefront of these developments that can be seen in the studies of the spatial structure and function of the city, urban human dynamics, urban cognition, urban metabolism, spatial economy, and many other topics under urban science (Batty 2021). Meanwhile, the availability of “big data” appears to be pushing us to demand even more data with our thirst for data stimulating new hypotheses and generating a new understanding of city systems where our need for new theory is beginning to outstrip our demand for new data. On the other hand, as we will elaborate in Sect. 3.1, new data and the accelerating development of cities pose new challenges to the usability of classical theories and analytical methods and this drives the momentum we seek to capture in this new journal.

### Urban sensing

Bridging the urban environment and urban analytics are urban sensing technologies for collecting data about both the physical environment and activities in the city. Apart from serving as a basis for urban analytics, the result of urban sensing can also directly contribute to decision-making or service delivery, with representative application fields such as environmental assessment, natural resource management, transportation, and disaster mitigation (Avtar et al. 2020).

Leveraging the latest sensing technologies, nowadays urban objects can be sensed from space and the sky, on the ground, and even underground and underwater, by using a wide range of sensors such as optical cameras and panorama cameras, synthetic aperture radar (SAR) and interferometric synthetic aperture radar (InSAR), laser scanner via light detection and ranging (LiDAR), ground-penetrating radar (GPR), and various sensors embedded in urban facilities which form an important aspect of the IoT (Shi 2021). Under the increasingly notable concepts and tools of social sensing, data sources such as massive cellphone tracking records and social media content have been also widely used in the analysis of urban function and activities. The collection of data such as cellphone tracking records can be seen as implicit crowdsourcing, which means that users may be unaware that they are crowdsourced. Explicit crowdsourcing, in which users voluntarily contribute content, is another important way of urban sensing. Those contributions related to locations or places are usually referred to as volunteered geographic information (Goodchild 2007), for example, crowdsourced maps (e.g., OpenStreetMap), geotagged photos (e.g., on Flickr), and online user reviews for locations and places (e.g., on Foursquare and TripAdvisor).

Besides the use of new data sources, other major trends in urban sensing include processing methods for data with very fine spatial and temporal resolution, as well as the use of AI and deep learning. While deep learning tends to outperform traditional methods of data processing for urban sensing, realizing such performance also tends to demand more labelled training samples, that is, more prior knowledge about the ground truth being sensed. Facing this challenge, unsupervised or weakly supervised AI models (e.g., by using crowdsourced training samples) have been studied to obtain more data-driven decisions (Shi et al. 2020).

### Urban big data infrastructure

Urban big data infrastructure includes not only urban big data and data platforms, but also the software and sometimes the dedicated hardware for realizing the infrastructure, and, further, the users of big-data products (Goodchild 2021b). Currently, shifting to the third dimension is one of the major responses of urban big data infrastructure to the growing complexity of the urban environment. The three-dimensional (3D) digital modeling techniques range from semantic 3D city modeling such as CityGML and building information modeling (BIM), to rule-based modeling such as CityEngine, and 3D management of property ownership. City-scale applications of CityGML and other 3D city modeling techniques have been realized in the areas such as business information service (Berlin Partner 2021), energy and climate (City of Helsinki 2022), and utility management (Zhang 2022). For transportation, the high-definition (HD) map is the key 3D data infrastructure for autonomous driving. HD maps not only enable self-navigation of the vehicles, but also provide detailed information in the vehicle’s surroundings which supports their decisions in self-driving, such as adjustment to cornering speeds.

A further response to such growing complexity is the integration of infrastructures, much in line with the transdisciplinary nature of urban informatics. The integration between BIM and geographical information system (GIS) is essential for the applications which require detailed data of both facilities such as buildings and their environmental context (Ma & Ren 2017). The latest version 3.0 of CityGML standard enables better incorporation of real-time IoT devices and more direct mapping of BIM to CityGML, paving the way for digital twins being employed for real-time urban management and applications in a seamless indoor-outdoor environment (Kutzner et al. 2020).

### Urban computing

The objectives of urban computing may be loosely divided into two types: one is to enhance the ability of computation, and the other is to obtain new or better results from methods, models, and tools for urban data, or what are called urban analytics. Both dimensions are indispensable for the data to create value for decision-making and services.

As a shared approach to computation capacity, cloud computing provides on-demand services at the software, platform, infrastructure, and other levels, enabling users to access high-quality computation resources at a lower price. Edge computing distributes the computation to the sensor network, which enables many previously unrealizable applications relying on both fast sensor response and computation power (Shi & Zhang 2021). Cloud and edge computing are also the backbones of AIoT which will be dealt with later in Sect. 3.

The proliferation of urban analytics includes methods for geographically rich data analysis and mining, of which some common aims are knowledge discovery or recommendation, as well as a great variety of urban simulation models, such as microsimulation, cellular automata, and agent-based models. Many AI and deep-learning approaches originally designed for non-spatial data have been adopted to the urban spatial context. For example, many studies have used transfer learning to recognize human activities from smart sensing data in different built environments (Cook et al. 2013). Generative adversarial networks (GAN) have been used to generate realistic movement trajectories and traffic flows, to help autonomous moving platforms to predict pedestrian motions (Gupta et al. 2018) or to predict traffic conditions (Zhang et al. 2019). The problem of opaque parameters, or the lack of interpretability of the AI methods, also stimulates research on explainable artificial intelligence (XAI) for urban computing tasks, such as for processing remotely sensed urban imagery.

### Urban applications

Available space–time data and corresponding tools and methods have enabled a vast variety of applications for planning and management of all kinds of subsystems in cities (Kwan 2021). Some subsystems emphasize improving the efficiency of the city and decision-making. Cities have implemented numerous platforms to optimize complex urban transportation systems, improve trip planning by citizens, and reduce congestion. Based on users’ trip and current locations, their likely interested places and activities may be inferred, so that customized recommendations and advertisements can be sent. Some other systems pay more attention to resilience and sustainability, such as to use advanced sensing and computing technologies to better respond to urban disasters, to prevent crime, to monitor pollution, and to reduce energy usage. Numerous studies on the relationship between human mobility and COVID-19 transmission (Zhang et al. 2022) provide other examples showing how public health may benefit from urban informatics.

As people increasingly rely on computational methods for the operation and management of urban systems, algorithms have become more important for decision-making. Algorithmic decision-making can take the form of augmenting human expertise with algorithms, using automatic systems in place of human decision makers, or using sophisticated optimizations which cannot be achieved easily by human brains. Algorithms can greatly save labor and improve system performance in terms of specified objectives, and they can potentially reduce individual biases. Yet at the same time, algorithmic decision-making can also enforce or even create new biases (Singleton & Spielman 2021).

## Research initiatives for urban informatics

### Developing a robust urban science

There are many theories about how cities are structured, how they evolve, and how they function. Many of these theories come from the mainstream social sciences but in parallel there is an emergent theory of physical evolution of cities in terms of their buildings, natural environments, and ecologies. In fact, it has been exceptionally difficult to develop integration between these many theories and they remain rather separate perspectives on the way we might understand cities and the ways in which we might plan and design their futures to meet sustainable development goals. Urban science as we have indicated above attempts to establish key features which can be generalized and pertain to many cities. Although in our domain it is not possible to establish laws of cities in any sense, there are well-defined long-term regularities which can be and are being captured using simple relationships such as power laws. Together with a long history of social physics, these developments suggest that urban science might lead to a more general theory of how cities function. That is the quest.

It will take an enormous effort to progress these ideas but urban informatics does establish a focus that could be critical in making progress in this kind of science. Currently new ways of looking at cities as economies, new views of mobility in the city from migration to local movement across many scales, new ways of thinking about the ecological structure of cities and the ways in which energy is mobilized, all of these suggest that urban science might begin to provide key insights into what we are able to do in realizing more sustainable cities in the future through ever-more-relevant interventions and planning. Because so much in urban science depends on flows between locations and over time, this perspective has great promise for developing an integrated approach to the many relationships that define the form and function of cities, and more particularly how the deep transition from a material to a digital world, from energy to information, might be embraced in developments of new urban theory. To date, most of our science deals with material and people flows; in the future it is information flows that will dominate if they do not do so already. In this sense, urban science opens a window into the virtual as well as the physical and social worlds.

During this current century, it is very likely that world population will stabilize and that many cultures will pass through the demographic transition. At the same time, the world is becoming digital and many activities such as retailing, education, parts of health care, and much manufacturing is moving online or becoming informed by new information technologies which themselves are online. This suggests that the basic relationships in urban science in terms of flows are also changing and evolving. Thus a robust urban science is critical to make sense of this emergent world, a science that will fashion plans for future cities that take all these kinds of changes into account. What cities will look like by the year 2100 is quite uncertain, for technologies are likely to change not only the way we move but why we move. There is the prospect of much more AI being used to build our cities and this could change what, where, and how we build for the new urban future. The sort of relationships that urban science is already working with will themselves evolve as cities are getting ever more complex and thus a robust set of ideas is sorely needed. We hope the journal will offer a platform for those working with this science to disseminate their ideas.

### Backbone technologies

#### GeoAI for fine-resolution remote sensing and urban social computation

Integrating geospatial science and AI, GeoAI (Janowicz et al. 2020) has been greatly enhancing the dynamic perception and knowledge discovery for geographical phenomena and earth science processes. Traditional machine learning relies on manual feature design which is limited by the domain knowledge of humans. Instead, deep learning has extraordinary ability in high-dimensional data processing and automatic feature extraction, which enables its full use of the increasingly available multisource and fine-resolution urban-sensing data to learn the complex natural and social processes and reach more intelligent decisions beyond humans’ previous knowledge.

##### GeoAI for fine-resolution remote sensing

In recent years, the development of smart cities has great benefited from the progress of remote sensing in spectral resolution, spatial resolution, temporal resolution, and the achievement of full-time and full-weather earth observation capability. With the assistance of high-performance computer hardware, the application of AI to fine-resolution urban remote sensing data has been fruitful in 3D reconstruction, data fusion, image classification, image retrieval, image understanding, object recognition, and change detection (Lian et al. 2020; Ma et al. 2019; Shi et al. 2020; Yuan et al. 2021). However, because of the continuous improvement in the temporal resolution of remote sensing data, even to real-time, and the increase in spatial dimensions from 2 to 3D, it becomes more difficult for AI models to learn robust and discriminative representations from the multi-temporal, multi-sensor, and multi-angle remote sensing data. The diversity of AI models and the lack of training samples are also challenges in practical applications. Massive training samples with accurate annotation are usually generated by human interpretation and field surveys, which are time-consuming and labor-intensive processes. To solve this problem, numerous attempts have been made at unsupervised and weakly supervised AI techniques, and impressive results have been generated over the past few years (Haut et al. 2018; Romero et al. 2015; Zhan et al. 2020). Although these new AI techniques alleviate the lack of samples to a certain extent, there is still large room for improvement. The volume, variety, and complexity of urban-sensing data will increase rapidly, especially with more available IoT data. This puts forward more demanding requirements for self-learning AI techniques.

##### GeoAI for urban social computation

What make a typical urban area are the intensive economic activities and social interactions within or generated by it, as distinct from rest areas on the planet. Therefore, most urban studies deal with social aspects which make urban informatics more challenging than natural science, due to the involvement of various human issues. Another related strand is computational social science, while urban informatics emphases more on urban and spatial context.

Many urban fields are attempting to use AI technologies to solve urban problems, such as traffic and transportation, land use planning, environmental protection, urban management, and public health (Cai 2021; Grekousis 2019). As discussed above, some work has been carried out based on remote-sensing data benefiting from AI image processing framework, models and algorithms. However, most of urban data are not imagery data. Statistical data, location-based sensor data including both point data and trajectory data, are other major components of urban data. To take advantage of AI technologies, one of the most basic research initiatives is releasing one or more urban datasets for the establishment of urban informatics, whose role is similar to that of ImageNet to computer vision research. The challenge is, however, that the dataset should include as many relations as possible to describe urban phenomena in a systematic manner. Although knowledge graph has been recognized as a pathway, addressing privacy issues and representing uncertainty and unpredictability related to human interactions make this task especially challenging. Consequently, exploring knowledge representation learning (KRL) for entities and high-dimensional relations among urban data is another critical task. The embedded representation of social-economic-environment knowledge could serve as an infrastructure to support the design of spatially- or urban- explicit AI models and algorithms, to gain deeper understanding of urban phenomena and processes. Establishing such datasets and their representation learning could contribute to both urban informatics and GeoAI.

#### Spatial data infrastructure and the HD map

The concept of a national spatial data infrastructure was originally promoted in the early 1990s (National Research Council 1993) as a solution to fundamental disruptions in the production of geospatial data, which until then had been dominated by agencies in higher levels of government. One of the most popular and important components of that infrastructure was the transportation map, including the network of streets and roads, canals and waterways, and railroads. Applications were emerging at that time that allowed users to plan routes through the network using wayfinding early versions of the tools that today are an essential part of modern life. These applications require a representation of the network that is navigable – that is, it includes details of permitted turns at intersections, overpasses and underpasses, one-way streets, and any other information that is essential for route planning.

The development of autonomous vehicles has added new requirements to the transportation layer. These vehicles will need much more information than is currently provided by the traditional databases: for example, to find, enter, and utilize parking structures, to find refueling facilities, to understand and respond to street signage, to maintain lane discipline, and to respect pedestrian crossings. Major investments are consequently being made in what have become known as high-definition (HD) maps, and in research into how best to acquire and represent the necessary information. These maps will need positional accuracies that are significantly better than that of current mapping, and spatial resolutions in the decimeter range or finer. Moreover updating presents a major challenge given the tendency for many of these feature types to change through time, and the poor state of tools for integrating geospatial data.

However an even more challenging problem concerns the relationship between centralized storage of geospatial data, which has been very much the model for spatial data infrastructure, and the advanced sensors that are essential features of autonomous vehicles: radar and LiDAR imaging and GPS. How should information available from central storage be integrated with the information being collected by the vehicle’s sensors? Here the analogy to the senses of the driver seems relevant: drivers navigate parking structures using their own sensors, so why should an intelligent autonomous vehicle not do the same? In effect the autonomous vehicle is a field robot, capable of using centralized data when needed but otherwise as fully functional as a human driver. Moreover sensed information is always up to date.

#### Quantum computing and quantum machine learning (QML)

Quantum computing is computation using laws of quantum mechanics (e.g., superposition and entanglement). By utilizing quantum behaviors, quantum computing can create very-high-dimensional spaces to solve many complex problems, especially simulation and optimization, many times faster than using classical means.

Real quantum computers typically comprise superconducting quantum processors, together with huge cooling systems to keep the hardware superconductive at a temperature barely above absolute zero. Although many quantum computing studies are conducted on real quantum hardware, the use of quantum circuit simulators on classical computers is also a common alternative.

Based on algorithms utilizing quantum phenomena and logic, QML has been proven to be dramatically faster and to achieve higher performance (e.g., accuracy) than its classical counterparts (Mishra et al. 2021). QML has covered supervised and unsupervised learning, and moreover, quantum neural networks have stimulated the field of quantum AI which is “still a much more debatable concept (Mishra et al. 2021, p. 126)”. A few large companies have lately launched cloud-based quantum computing resources: for example, Google’s Tensorflow Quantum machine learning framework, Microsoft Azure Quantum Preview, and IBM Quantum and Qiskit, making quantum computing and QML widely accessible to researchers and developers.

Since quantum algorithms have become widely evaluable on available hardware only for the last few years, the latest QML research still focuses on the development of theories and algorithms for fundamental machine learning tasks (e.g., general classification and image processing). Industrial applications of QML concentrate on fields such as materials science, physics, and finance (IBM 2022). Although the suitability for quantum computing to solve large-scale transportation problems has been figured out, real-world QML applications to modeling and optimization of transportation or other urban systems are very rare. Yet like other machine-learning technologies, the fundamental QML algorithms may be extended to urban and geospatial studies soon. More excitingly, in view of the physical principles utilized in the research of complex urban systems, more specialized QML for these systems may emerge, where the basic elements in the systems might be modeled by means similar to quantum molecular simulations for physical sciences.

#### AIoT and city digital twins

As an integration of AI and IoT, AIoT allows each device in the IoT (i.e., the edge device) to have its own AI which can realize smart applications and communicate with other AIs in the network. AIoT is currently one of the most versatile technologies in urban informatics. It enables AI tasks that were previously computational expensive, from image and video analytics, text and voice recognition, to biometric recognition and human pose estimation (Zhang & Tao 2021), to become available on mobile devices and smart sensors. As a result, a lot of latency-sensitive and intelligence-demanding applications, such as autonomous driving, self-navigation of robots and UAVs, elderly fall detection, and smart security systems, are either enabled or greatly enhanced. While centralized computing alone could be powerful for urban management and service delivery, AIoT would provide a foundation for an urban environment with “complete ambient intelligence”, resulting in greatly improved efficiency and convenience.

A major challenge in AIoT is to design lightweight and efficient deep-learning models which work well on edge devices with limited computation capacity. To obtain such models involves multiple techniques under intensive research, including network pruning, compression, and quantization. Some other difficulties are the scarcity of labeled samples and the requirement that deep-learning models be adaptable to the diverse urban contexts that edge devices are facing, and the wear and tear on the edge devices themselves. To solve these difficulties, machine-learning strategies such as self-supervised learning, zero-shot learning, transfer learning, and domain adaptation have been recently studied in the AIoT context (Zhang & Tao 2021). Also, AIoT devices have access to a lot of private data at the individual level. The technologies for AIoT to perform AI while protecting the users’ privacy and data security will be discussed in Sect. 3.2.7.

A digital twin is a virtual digital representation which serves as a mirror of a physical system. In the ideal case, by conducting detailed sensing and computing, the status and changes of the physical system can be accurately copied in real time in the digital twin. Digital twins can be used for visualization, monitoring, diagnostics, operation, and simulation of systems, and they have been applied to manufacturing, construction, healthcare, the automotive industry, and also fields more pertaining to urban systems, such as urban planning and traffic optimization.

AIoT can serve as a core technology of digital twins and greatly improve their function: densely embedded sensors can obtain real-time data from the physical system, and efficient AI models running on both edge devices and the cloud can achieve real-time representation and decision-making. To date, the integration of AIoT and digital twins has mainly focused on smart industry and manufacturing (Jin et al. 2020; Yu et al. 2021). Fine-resolution digital twins at the city level, or “city digital twins”, especially those leveraging AIoT, are still in their infancy. The common current form of the city digital twin is the city information model (CIM) which fuses GIS and BIM (Cureton & Dunn 2021). The latest digital twins may be able to predict the physical state of the city, but social phenomena and human behaviors which influence the prediction remain very challenging to be precisely incorporated. Thus, some scholars regard the comprehensive city digital twins, especially in the social perspective, as unlikely to be realizable in the near future (Cureton & Dunn 2021).

Nevertheless, some latest AIoT technologies are capable of capturing human behaviors, including analyzing human emotion and mental status from facial expression and eye movement, obtaining detailed human physical status by using all-around wearable electronics based on triboelectric nanogenerators (TENGs), and AI-based extraction of individual activities from mobile device tracking data. While the objectives of these sensing tasks are still much simpler than people’s social behaviors, the above technologies may pave the way to better simulation and prediction of social behaviors in future city digital twins.

#### Explainable artificial intelligence (XAI)

Data-driven AI models, represented by deep learning, enable automatic and intelligent processing of urban-sensing data and urban analytics. But the highly complex deep-learning models usually have low transparency, which brings difficulties to interpret the decisions of such models, resulting in a reduction of trustworthiness that severely limits their further applications. Also, data portions or automatically extracted features that actually represent biases or discrimination (e.g., implying a certain ethnical group or disadvantaged group) may make significant contributions to the AI-based decision-making without the knowledge of human users.

XAI aims to discover the decision-making processes of specific AI models and provide interpretable prediction for human users. Following years of development of XAI in classical machine-learning tasks such as image, video, and natural-language processing, a few studies have started to deal with XAI for remote sensing (Arun & Karnieli 2021; Dikshit & Pradhan 2021). There is even less research on XAI for urban analytics which are further away from the classical machine-learning tasks, especially for the tasks that heavily involve the less predictable human social behaviors. However, XAI for urban analytics is likely to develop rapidly in the near future. XAI has a high potential to promote a new generation of urban analytics with high intelligence and interpretability, thus the diagnoses can be done to improve the model reliability, the risks associated with the uncertainty of model predictions can be reduced, and potential biases and discrimination may be better spotted and removed to increase the equity and fairness of algorithmic decision-making.

#### Privacy-preserving deep learning and distributed machine learning

Concern for data security and personal privacy has been increasing together with the fast-growing capacity of urban sensing and the fine spatiotemporal resolution of the resultant data. For example, IoT devices can obtain individuals’ biometric data, movement trajectories, and information about their living environments, and individuals’ activities can be recognized from fine-resolution remote sensing. For this issue, urban computing technologies, especially AI, have been developed to learn in a privacy-preserving way. As a current mainstream solution for privacy-preserving deep learning, federated learning (FL) is a distributed machine-learning framework that enables joint modeling by using data from several parties without the need for them to disclose their own raw data. An alternative is split learning (Gupta & Raskar 2018), which splits the deep neural network into the parts (layers) on the side of multiple data sources and the part on the server side, thus realizing the training without passing data to the server. Apart from many applications of FL in AIoT, specific FL architectures have also been built for remote-sensing data processing (Tam et al. 2021) and location-based services (Huang et al. 2021).

FL and other distributed machine-learning frameworks, whether privacy-preserving or not, have multiple other benefits for processing urban-sensing data. From the perspective of AI algorithm implementation, urban-sensing applications usually face challenges of large data volume, multiple data formats, and complex scenarios, which make the algorithms complex, difficult to understand, and computationally expensive. Complex tasks which make it difficult for a single AI algorithm to achieve good performance can be decomposed into multiple simpler and easy-to-understand tasks, and their modular processing can be realized by using distributed computing. This decoupling implementation may not only improve the execution efficiency and reduce the cost of massive data transmission, but also help to enhance the interpretability and reliability of the algorithms.

While existing FL studies have achieved admirable training results, a number of implementation challenges remain to be solved. In particular, FL can suffer from lengthened latency due to slow devices participating in the training and communication instability (Lim et al. 2020). More research is needed to improve large-scale implementations of FL in highly latency-sensitive applications, such as autonomous driving.

### Towards future cities and citizen lives

#### Urban design and planning: issues, the science, and transdisciplinary perspective

In the years ahead, existing contemporary urban issues may remain central topics in urban design and planning, particularly those related to continuous urbanization, such as job-housing balance, congestion reduction, urban renewal and relocation of the usually vulnerable original residents, planning of urban agglomerations and vertical cities, and to promote the livability of the continuously aging population. Understanding of these issues will likely be deepened, and their computational solutions sharpened, by the latest technologies and tools in urban informatics. Meanwhile, new changes to cities and urban people behaviors, such as those discussed in Sect. 3.1, will undoubtedly augment the set of essential issues for urban design and planning. More sensing and computing technologies and tools specialized for these new issues are likely to emerge in an accelerated manner, and the decision-making process will likely be more data-driven.

As the sectors in cities become more specialized, it is increasingly necessary and complex for city design and planning to coordinate contradicting perspectives from different sectors. A simple example is that during COVID-19, many cities promote cycling as well as outdoor dining to reduce close contacts and infections. Contradiction occurs when the extended outdoor dining areas get very close to or even take over the bike lanes, causing traffic accidents and unavailability of bike lines for the growing number of cyclers. Worse still, the narrowed walkable space due to outdoor dining and sometimes also cycling can greatly increases the difficulty for the elderly and disabled to move around, aggravating the social exclusion problem (Bou Akar, 2021). As explained in Sect. 2.1, urban informatics provides the chance to tackle such complexity with rich urban-sensing data, high-dimensional models, and multi-objective optimizations, to reach a better compromise between different urban sectors and stakeholders.

#### Transport: connected, autonomous, sustainable, and shared

Supported by AIoT and many other urban sensing and computing technologies, the future transport envisioned by many people will achieve full connection among vehicles, infrastructure, pedestrians, and the network via Cellular-V2X. Sustainable transport, such as higher-performance electric vehicles and improvement of urban walkability, as well as shared passenger transport and logistics, are already on the agenda. The interest in high-speed transportation will likely continue, as exemplified by hyperloop, a form of mass transit within near-vacuum tubes by using magnetic or aerodynamic propulsion which can achieve double the speed of airliners (Motwani & Gupta 2021).

New transportation technologies will profoundly influence the city’s morphology and logic of function organization (Wang 2021), since the distribution of urban activities strongly depends on the means, speed, and cost of the travel between and during these activities. Similar to planning, future transportation systems would greatly benefit from transdisciplinary perspectives. Such perspectives will integrate new land use and urban activity patterns due to the digital transformation and newly involved urban systems (e.g., AIoT and renewable energy sources), to promote the efficiency of cities and, hopefully, also equity in the mobility and accessibility of different social groups.

#### Location-based services (LBSs) and geoprivacy

Thanks to positioning and sensing technologies, together with powerful algorithms, LBSs are bound to become more efficient and powerful. Door-to-door navigation will be available on regular smart phones, guiding users to travel seamlessly in a mixed indoor and outdoor environment. Mobile devices supporting extended reality (XR) technology will provide people with metaverse experiences, which will be further envisioned in the next subsection. The services will be more customized and better fulfill the needs of special groups. Location-based advertising will become more accurate and profitable. More navigation applications or other LBSs tailored to people with limited mobility or cognitive abilities will emerge, helping them to more easily access the resources needed to sustain their quality of life and reduce their disadvantages compared with the “standard users”. People’s health and safety may be better protected by affordable wearable devices with more accurate positioning, posture estimation, and health monitoring in different scenarios such as construction and health care. The devices will help more people with chronic illness get rapid treatment during attacks, and more children may be forestalled from falling from high buildings or be quickly located once kidnapped.

The higher utility of LBSs is usually accompanied by the finer spatiotemporal resolution of location information and rising level of privacy intrusion (Keßler & McKenzie 2018). The intrusion can be more threatening when combined with other user data collected by the multifunctional LBSs, such as biometric data, personal financial data, and social relationships. The morality of many LBSs is still under debate, and even for the accepted ones, there can be considerable risks for the service providers to abuse the collected data, and for users to abuse the services. For example, controlling parents may monitor every move of children when they are out of sight through kid’s smart watches. There are pressing needs for laws and standards regarding geoprivacy and protection of the potential affected groups to match the fast-rising capacity of LBSs.

#### Metaverse and prospective MetaSocieties

“Metaverse” is usually the term for the gigantic, decentralized, and shared immersive Internet environment fusing the physical and digital worlds that covers all aspects in people’s lives (Lee et al. 2021). The metaverse has attracted many investigations and technical proposals for over a decade, in fields such as retailing (Bourlakis and Papagiannidis 2006), education (Kemp & Livingstone 2006), and collaborative research (Forte & Kurillo 2010). The topic has become significant again since its enabling technologies, including XR, AIoT, edge computing, and blockchain, became widely available.

The growth of the metaverse has been expedited by the demand for being contactless during COVID-19, through the prevalence of online meeting, virtual tourism, virtual fitness classes in which one can learn together with real-time holographs of the instructor and classmates, and likewise activities. Some scholars predict that the expansion of human activity space by the metaverse will lead to “MetaSocieties” in parallel with physical societies, in which humans, enterprises, and even cities will all have their virtual counterparts. The changes of the MetaSocieties and physical ones will affect each other (Wang et al. 2022).

The enabling technologies of the metaverse need to be further developed, particularly to become more accessible and affordable, to implement the metaverse on a wide scale. This is the chance to advance the technological base of urban informatics, since these enabling technologies are to a large degree about urban sensing, computing, and big data infrastructure. Moreover, the metaverse and overall trend of digitalization will undoubtedly elevate the importance of the principles in the virtual society and physical-virtual interactions in future urban science, planning, and analytics. Geo-social studies will also face the complexity introduced by the metaverse counterparts of many geo-social issues, such as (geo)privacy, equity to diverse users, and cyberbullying (Lee et al. 2021). These would be arduous research tasks, considering that the technological revolutions in the last decades have been usually ahead of the social progress needed to rein in the new social problems generated.

#### Algorithmic and data-driven approaches: representativeness, equity, and uncertainty

Urban decision-making and services in various sectors of cities will be inevitably more algorithmic (i.e., based on computational algorithms) and data-driven. While enjoying the higher efficiency, cost-saving, and stronger functionality of the algorithmic and data-driven approaches, being aware of the unintended consequences becomes newly important. Algorithmic and data-driven approaches may potentially reduce human subjectiveness and biases, but they can also enforce or even create new ones. As explained in Sect. 3.2.5, AI can learn implicit biases or discrimination in data and replicate them in subsequent decision-making. XAI helps discover the biases or discrimination, but to correct for them is far from straightforward.

One difficulty is that completely unbiased datasets appear to be unreachable, especially for the user-generated ones which nevertheless have growing importance. Online user-generated content has been recognized as representative of such difficulty, but lately they seem to be joined by AIoT. For example, FL on IoT data tends to select participating devices with higher computation capacity to ensure the overall learning performance. Thus, users with better devices will be overrepresented and more likely well served by the learning results. Fortunately, scholars have started to tackle this fairness issue (Lim et al. 2020). The quality and bias assessments of user-generated urban big data have been advocated for years (Antoniou & Skopeliti 2015; Senaratne et al. 2017). Of similar importance is to develop the methods to rectify the analysis results despite data biases. Due to the complexity and involvement of social behaviors of such data, current developments of their bias assessments or rectifications are far from meeting people’s eagerness to become more algorithmic and data-driven.

Another urgent need is to evaluate the (un)predictability within the models and establish the accountability for the predictions, especially for the urban sectors that significantly involve humans. Models in such sectors usually need to either explicitly consider human behaviors or make assumptions about them. For example, for facility allocation planning, the preference of different population groups for different facilities may need to be involved. For traffic optimization, the drivers’ tendency to choose the predicted fastest routes, leading to a shift of congestions to those routes, will need to be considered. While more aspects in the city can be sensed and predictively modeled in great detail by using powerful technologies including AIoT-facilitated city digital twins and even the metaverse, human social behaviors that are critical to the actual outcome concerned by the predictions remain “less predictable than assumed” (Cureton & Dunn 2021, p. 269). When the predictions on the counterpart of an individual in a powerful simulation, a digital twin, or the metaverse are so detailed that they look very much persuasive, what will be the consequences if the predictions are inappropriate? Decisions made by the predictions may increase the physical and mental difficulty for the person to make other choices, and thus the person is actually driven to the prediction results. If the consequence of such a situation is harmful but not immediate, who will be responsible for it? Let alone that people underrepresented by the models and consequently suffering from inappropriate predictions will continue to be more likely those disadvantaged groups, such as people with low income, less access to the Internet, or limited mobility. Disastrous consequences due to people’s excessive faith in technology are no longer fictional.

## Concluding remarks

This article has introduced the motivation behind the launching of the *Urban *Informatics journal, briefly reviewed the development of urban informatics, and presented a collection of research initiatives which comprise the field. These initiatives first covered the new urban sciences, followed by the development of core technologies such as GeoAI, HD map, quantum computing and QML, AIoT, city digital twin, XAI, and privacy-preserving deep learning in the urban informatics context. Then the initiatives were proposed in terms of the applicational demands and broader effects on cities and citizen lives, including the perspectives of urban design and planning, transport, LBSs, and metaverse, and finally some discussion of algorithmic and data-driven approaches.

Due to the immense scope of urban informatics, the initiatives proposed and listed in this short introduction are bound to address only a very limited part of the whole. The authors were unable to cover many of the enabling technologies for the applications-based developments mentioned in Sect. 3.3. For example, the technologies underpinning each new mobility mode listed in Sect. 3.3.2 would require extensive reviews and proposals for research initiatives. Sustainability and resilience are other major topics: for instance, how to realize carbon neutrality more smartly and with less economic cost, how GeoAI can enable more effective disaster mitigation, and how future cities may be more resilient to pandemics which are expected to become more frequent in the coming century (Marani et al. 2021). As the urban population becomes more reliant on information technologies, the cost of failures or malicious uses of the technologies will grow in proportion. For this issue, data and network security also deserve much more discussion.

The technologies and issues dealt with in this introductory paper are so interrelated that it is somewhat difficult to distribute them into clearly different subsections. This exactly exhibits the interdisciplinary nature of urban informatics. Many reviews on specific technologies or issues related to this field, such as those cited in this article, also mention such interrelations between the objectives of the reviews and other technologies and issues. However, this young field needs more transdisciplinary viewpoints on the macroscopic technological transformation and broader implications. The authors look forward to more reviews and comments addressing this topic.

Those concerns aside, it will have become clear to the reader of this introduction that urban informatics presents a useful and important basis for scholarship, and an important framework for research into the future of cities. As with all such discussions at the intersection between technology and society, it raises an important concern: how should the push towards ever-increasing reliance on technology be balanced with societal concerns about ethics, equity, and inclusion? How can we ensure that future developments in urban informatics address issues of importance not only to the physical structure and design of the city and its efficiencies, but also to the needs and concerns of the city’s population? The authors believe that the future of this journal will depend very much on achieving the right balance.

## Data Availability

The article has no associated data.

## Abbreviations

3D: Three-dimensional
AIoT: Artificial intelligence and the internet of things
BIM: Building information modeling
CIM: City information model
FL: Federated learning
GAN: Generative adversarial networks
GeoAI: Geospatial artificial intelligence
GPR: Ground-penetrating radar
HD map: High-definition map
InSAR: Interferometric synthetic aperture radar
KRL: Knowledge representation learning
LBS: Location-based service
LiDAR: Light detection and ranging
QML: Quantum machine learning
SAR: Synthetic aperture radar
TENG: Triboelectric nanogenerator
XAI: Explainable artificial intelligence
XR: Extended reality
